# Incidence and Prevalence Analysis of Non-Small-Cell and Small-Cell Lung Cancer Using Administrative Data

**DOI:** 10.3390/ijerph18179076

**Published:** 2021-08-28

**Authors:** Andrea Ricotti, Veronica Sciannameo, William Balzi, Andrea Roncadori, Paola Canavese, Arianna Avitabile, Ilaria Massa, Paola Berchialla

**Affiliations:** 1 Department of Public Health and Pediatric Sciences, University of Torino, 10100 Torino, Italy; andrea.ricotti@unito.it; 2 Unit of Biostatistics, Epidemiology and Public Health, Department of Cardiac, Thoracic, Vascular Sciences and Public Health, University of Padova, 35121 Padova, Italy; veronica.sciannameo@unito.it; 3 IRCCS Istituto Romagnolo per lo Studio dei Tumori (IRST) “Dino Amadori”, 47014 Meldola, Italy; william.balzi@irst.emr.it (W.B.); andrea.roncadori@irst.emr.it (A.R.); 4 Roche S.p.A, 20900 Monza, Italy; paola.canavese@roche.it (P.C.); arianna.avitabile@roche.it (A.A.); 5 Department of Clinical and Biological Sciences, University of Torino, 10100 Torino, Italy; paola.berchialla@unito.it

**Keywords:** non-small-cell lung cancer, small-cell lung cancer, incidence

## Abstract

Treatment of lung cancer depends on the stage of the tumor and the histological type. In recent years, the histological confirmation of lung non-small-cell lung cancer has become crucial since the availability of selective target therapeutic approaches. The aim of the study was to develop a validated procedure to estimate the incidence and prevalence of non-small-cell and small-cell lung cancer from healthcare administrative data. A latent class model for categorical variables was applied. The following observed variables were included in the analysis: ICD-9-CM codes in the Hospital Discharge Registry, ATC codes of medications dispensed present in the Drugs Prescriptions Registry, and the procedure codes in the Outpatient Registry. The proportion of non-small-cell lung cancer diagnoses was estimated to be 85% of the total number of lung cancer on the cohort of incident cases and 89% on the cohort of prevalent cases. External validation on a cohort of 107 patients with a lung cancer diagnosis and histological confirmation showed a sensitivity of 95.6% (95%CI: 89–98.8%) and specificity of 94.1% (95%CI: 71.3–99.9%). The procedure is an easy-to-use tool to design subpopulation-based studies on lung cancer and to better plan resource allocation, which is important since the introduction of new targeted therapies in non-small-cell lung carcinoma.

## 1. Introduction

The Italian Association of Medical Oncology estimated 42,500 new lung cancer cases in Italy in 2019. Of them, 29,500 (69%) occurred in men and 13,000 (31%) occurred in women. The lifetime odds of developing lung cancer are 1:11 for men and 1:39 for women [1]. According to the WHO classification, more than 95% of lung cancers can be traced back to two main histotypes: non-small-cell lung cancer (NSCLC) and small-cell carcinoma (SCLC) or microcytoma [2]. In turn, NSCLC comprises three main subtypes: squamous cell carcinoma, adenocarcinoma, and large cell carcinoma.

Treatment of lung cancer depends on the histological type and the stage of the tumor, and in recent years, the histological confirmation of NSCLC has become crucial since the availability of selective target therapeutic approaches [3]. Thus, it is important to estimate the incidence and prevalence of histological lung cancer subtypes for better planning the resource allocation needed for the management of the disease [4,5].

In Italy, about 75% of the lung cancer cases collected by the cancer registries have histological confirmation (data referring to years 2010–2014): Of these, 40% are adenocarcinomas, 21% are squamous carcinomas, 12% are small-cell tumors, and 2% are large-cell tumors, whereas the remainder is other unspecified morphologies [1]. The diagnosis of NSCLC is typically based on morphological evaluations of biopsy or surgical specimens [3,6]. In SCLC, the diagnosis is mainly based on nuclear characteristics of a cytological sample [7].

Current healthcare administrative data represent a valuable source of information for Real-World-Evidence planning resources allocation and carrying out population studies [8]. The ICD 9th Revision, Clinical Modification (ICD-9-CM) codes in the Hospital Discharge Records, outpatient procedures codes, and the Anatomical Therapeutic Chemical (ATC) classification of medications are widely used to select study cohorts [9]. However, these data sources lack sufficient granularity for specific subpopulation studies. Such limitation is apparent in subpopulation studies on lung cancer, for which, for example, there is not an ICD-9-CM code specific for non-small-cell lung cancer and small-cell lung cancer diagnosis [10].

So far, no Italian studies have been published reporting the accuracy of using administrative data to identify NSCLC and SCLC cases. However, some experiences assessing the validity of ICD-9-CM codes for lung cancer have been reported [9]. Several studies have been published in other countries, especially in US, where claims data, being primarily used for reimbursement purposes, are detailed and reliable. In a 2009 study, Ramsey et al. [11] estimated an accuracy of 51.1% in identifying incident NSCLC cases from administrative data using only ICD-9-CM codes.

Duh et al. [12] developed an algorithm to identify SCLC cases based on the treatment guidelines of the American Cancer Society and the National Comprehensive Cancer Network. The algorithm initially aimed to identify patients with SCLC; a modified version was subsequently proposed for identifying patients with NSCLC. In the latter algorithm, procedures and chemotherapies for patients with NSCLC are employed as inclusion criteria, while the exclusion criteria consist of chemotherapy regimens administered to patients with SCLC [12,13,14]. A recent validation of the algorithm estimated an accuracy of 92.1% with a sensitivity of 94.8% and a specificity of 81.1% [13].

More recently, reporting the results of the VALIDATE-J study, de Louise et al. [14] demonstrated high specificity and positive predictive value for claims-based algorithms in identifying both incident and prevalent lung cancers cases using Japanese claims data. However, the authors did not provide any consideration on differentiating SCLC and NSCLC.

Within the available literature in Europe, worthy of note is an incidence study carried out to estimate the cost of NSCLC treatment in France, Germany, and the United Kingdom [10]. In this study, patients diagnosed with NSCLC were identified from hospital discharge records, outpatient visits, pharmaceutical services; however, a validation of the procedure is lacking. This study was aimed at defining a validated procedure to estimate the incidence and prevalence of NSCLC and SCLC from healthcare administrative databases generated in routine care in a region of northwest Italy of about 43,000,000 residents [15] with 3450 incident cases in 2018 [16]. External validation of the results is also provided by assessing the accuracy, sensitivity, specificity, and the predictive values of the method on a clinical cohort of patients available at the Romagnolo Scientific Institute for the Study and Treatment of Tumours, in the region of Emilia Romagna, Italy.

## 2. Materials and Methods

### 2.1. Data

The study population included all residents in Piedmont, a northwestern region of Italy, during 2013–2017. Data were drawn from the Piedmont Health Information System, including the Hospital Discharge Registry, Drugs Prescriptions Registry, and Outpatient Registry. Cases of lung cancer were retrieved from the Regional Hospital Discharge Registry, according to the ICD-9-CM. Data on vital status were also obtained from the regional mortality registry.

A cohort of incident cases and a second cohort of prevalent cases were retrieved. To identify incident cases, we selected the records of patients with diagnosis of lung cancer between 1 January 2017 and 31 December 2017 using ICD-9-CM codes 162.X and 213.2 located in the primary position of the Hospital Discharge Registry; prevalent cases, i.e., patients with the same diagnosis (ICD-9-CM codes 162.X in any position) between 2013 and 2016 were excluded. The second cohort of prevalent cases was selected using ICD-9-CM diagnostic codes (162.X) in any position between 2013 and 2017.

The selected records were linked to the Drugs Prescriptions Registry and the Outpatient Registry.

From the Drugs Prescriptions Registry, records with the chemotherapy treatments listed in Table 1 were retrieved.

From the Outpatient Registry, information on radiotherapy, chemotherapy, and diagnostic procedures listed in Table 2 were retrieved. From the Hospital Discharge Registry, radiotherapy and chemotherapy procedures received were retrieving from ICD-9-CM procedure codes in any position (Table 3).

### 2.2. Statistical Analysis

NSCLC/SCLC classification of lung cancer histological type was performed using a latent class statistical model. Latent class analysis is a multivariate categorical data analysis technique that identifies and characterizes clusters of observations sharing a similar distribution of the variables of interest [18]. Latent class models attempt to stratify observations based on variables (manifest variables) crossed with an unobserved outcome (latent variable). Such models probabilistically assign each observation to a latent class based on the observed values of the manifest variables.

The following manifest variables were considered: (i) the type of chemotherapy treatment (since potentially, each patient has more than one chemotherapy treatment, the two most recent treatments were picked up) identified on the basis of the codes shown in Table 1; (ii) outpatient procedures listed in Table 2; (iii) having had a procedure among those listed in Table 3 (interventions on the respiratory system column); (iv) having undergone a radiotherapy treatment (Table 2 and Table 3).

Since the unobserved latent variable (the histological type NSCLC/SCLC) is a nominal variable, the latent class model is a finite mixture model.

Each of the 4 manifest variables considered has $k_{m}\;\left(m=1,\ldots ,4\right)$ categories. Let $Y_{imk}$ be the observed m−th manifest variable on the i−th subject such that $Y_{imk}=1$ if the i−th patient has the category k and 0 otherwise. For example, if the manifest variable is the type of chemotherapy treatment (m=1), the categories are 16 (i.e., the number of the ATC codes in Table 1) and therefore, k takes integer values between 1 and 16. Thus, k=2, $Y_{i12}=1$ if the *i*-th patient has taken the drug L01XE36 (the order in which the chemotherapy treatments are considered is irrelevant) and 0 otherwise.

The latent class model approximates the joint distribution of the manifest variables as the weighted sum of a fixed number R of mixture components. The number R, which is the number of latent classes, is selected a priori, and it can be determined based on theoretical considerations or based on the goodness-of-fit indices of the model. In our case, we set two latent classes based on the classification problem of dividing the lung cancer population into NSCLC and SCLC. Then, we evaluated the consistency of this assumption with the calculation of the Akaike Information Criterion (AIC) [19].

Let $\pi_{jrk}$ be the conditional probability that a patient in the latent class r (with r = 1, 2) has the k−th value on the j−th variable and let $p_{r}$ be the unconditional probability that a patient belongs to the r−th class before observing the manifest variables, i.e., the a priori probability latent class membership. Assuming the conditional independence between the manifest variables and the latent class,

(1) $$P\left(Y_{i}|\;\pi ,p\right)=\overset{R}{\underset{r=1}{\sum }}p_{r}\overset{J}{\underset{j=1}{\prod }}\overset{K_{J}}{\underset{k=1}{\prod }}\pi_{jrk}^{Y_{ijk}}$$

where $Y_{i}$ represents the probability density function of the manifest variables across all latent classes, and $p_{r}$ and $\pi_{jrk}$ are the model parameters that needed to be estimated. Given the estimates of the model parameters, the posterior probability that each patient belongs to each class, given the evidence of treatment and outpatients procedures, can be computed using Bayes’ formula.

The model parameters were estimated using the Expectation–Maximization (EM) algorithm [20]. The EM algorithm was chosen due to the iterative nature of the algorithm itself, which allows estimating the parameters of the model even in the presence of missing observations. In this case, in fact, the a priori probabilities are updated at each iteration using only the manifest variables for which observations are available.

All analyses were conducted with R version 4.0.2 (R Core Team, Vienna, Austria) [21].

## 3. Results

In Table 4, the description of the cohort of patients with a diagnosis of lung cancer between 2013 and 2017 is reported stratified for vital status at the end of 2017.

The number of incident cases in 2017 is 3472.

Overall, 2694 (77.6%) subjects underwent chemotherapy.

The interventions on the respiratory system listed in Table 3 were reported on the Hospital Discharge Registry for 1818 (52.4%) patients.

Finally, the 3472 incident cases were associated with 35,729 outpatient procedures, whose codes are listed in Table 2.

The number of prevalent cases in the years 2013–2017 was 5820. Overall, 4824 (82.9%) of them were chemotherapy.

### 3.1. Latent Class Analysis

To estimate the size of the two subpopulations (NSCLC and SCLC cases), a model with two latent classes was applied, both to the incident and prevalent cases, considering the following manifest variables: (i) the type of chemotherapy treatment (since potentially, each patient has more than one chemotherapy treatment, the two most recent treatments were considered) among those shown in Table 1; (ii) the outpatient procedures that patients underwent among those listed in Table 2; (iii) having had a procedure among those listed in Table 3 (column Interventions on the respiratory system); (iv) having undergone a radiotherapy treatment among those listed in Table 2 of the outpatients procedures and Table 3 (column Radiotherapy treatment).

Based on the AIC, which measures the goodness of fit of the data (the smaller the value, the better the model), the model without radiotherapy treatment is to be preferred in estimating the size of the two subpopulations, both for the incident and prevalent cases.

Regarding the incident cases, two latent classes comprising 15.2% and 84.8% of cases were identified (SE ± 1.1%) (Table 5). By examining the unconditional probabilities reported in Supplementary Table S1 for incident cases, the two subpopulations were assigned the type of lung cancer (NSCLC or SCLC). In fact, observing the ATC codes, class 1 mainly consists of patients treated with Etoposide (L01CB01, 33%), Carboplatin (L01XA02, 38%), Cisplatin (L01XA02, 23%), and residually with Topotecan (L01XX17). Moreover, class 1 also has a greater likelihood of outpatient procedures. Thus, SCLC cases were assigned to class 1 (15.2% of cases), whereas NSCLC cases were assigned to class 2 (84.8% of cases). For the prevalent cases, two classes comprising 10.9% and 89.1% of cases were identified (SE ± 1.1%) (Table 5). Following the same reasoning as for the incident cases, SCLC cases were assigned to class 1 (10.9% of cases), whereas NSCLC cases were assigned to class 2 (89.1% of cases).

### 3.2. External Validation

A first validation of the estimate of the size of the two subpopulations of patients with lung cancer (NSCLC and SCLC) was made by comparing the results obtained with the estimates reported in the literature. The most recent publication in which data are reported is the work of Mazzanti et al. [22], who cites the data in Carrato et al. [23] and Herbst et al. [24], for which the cases of NSCLC account for approximately 85% of lung cancers. As for the Italian population, the incidence figure of lung cancer is not presented stratified in the two forms NSCLC and SCLC [1].

The data from the Piedmont Region Cancer Registry reported 3450 incident cases in 2018, which was comparable with the data that was extracted from the administrative databases of 3472 incident cases in 2017 [25].

The most recent data available with cytohistological confirmation are those relating to the period 2008–2012 [19]. Cytohistological confirmation is present in about three-quarters of the series, and 12% of these are small cell tumors. These data are also compatible with the estimate of the prevalent cases (Table 5) equal to 10.9% (SE = 1%).

A cohort of 107 individuals resident in Emilia Romagna diagnosed with lung cancer at the Romagnolo Scientific Institute for the Study and Treatment of Tumours, in the region of Emilia Romagna (Italy), for whom cytohistological confirmation was available, was used as the gold standard to assess the sensitivity, specificity, and predictive values of the method developed. Overall, 87 out of 107 presented NSCLC. The model developed assigned to each patient a probability of membership to the latent class NSCLC or SCLC. Thus, the patients were assigned to the most likely (higher probability of membership) latent class.

Comparing the classification provided by the model with the gold standard, the overall accuracy was 95.3%, 95% Confidence Interval (95%CI): 89.4–98.5% (102 patients out of 107 correctly classified); the sensitivity was 95.6% (95%CI: 89–98.8%), and the specificity was 94.1% (95%CI: 71.3–99.9%). Finally, positive and negative predictive values were 98.9% (95%CI: 92.8–99.8%) and 80% (95%CI: 60.1–91.3%), respectively.

## 4. Discussion

To estimate the proportion of NSCLC and SCLC cases, we analyzed a cohort of incident cases of lung cancer between the years 2013 and 2017 in a region of northern Italy, and we applied a latent class analysis. The same analysis was repeated on the prevalent cases.

Overall, a proportion of NSCLC diagnoses was estimated to be 85% of the total number of lung cancer on the cohort of incident cases and 89% on the cohort of prevalent cases.

The estimate was obtained using a model with two latent classes, thus neglecting a potential 5% (according to estimates reported in the literature) of cases not classified neither as NSCLC nor SCLC histotype.

The radiotherapy treatment seems not to increase the information needed to discriminate between the two types of cancer. Improving the temporal setting of analysis of chemo or radiotherapy procedures (for example, by identifying the first, second, and third lines of treatment) would probably increase the discriminating power of such information.

Finally, we conducted an external validation of the model results in two steps. First, we compared the data obtained with the data published by the Piedmont Cancer Registry and published. The data from the Piedmont Region Cancer Registry confirmed 3450 incident cases in 2018, which is comparable with the 3472 cases in 2017 we retrieved from the healthcare administrative databases, showing a reliable selection of the cohort. A major limitation is given on the availability of data with cytohistological confirmation, which is currently available only on about 75% of cases diagnosed between 2008 and 2012. Nevertheless, 12% of these cases are confirmed SCLC cases, which is compatible with the estimate of the prevalent cases (Table 5) equal to 10.9% (SE ± 1%).

Second, we conducted a validation on a cohort of patients diagnosed with lung cancer, at the Romagnolo Scientific Institute for the Study and Treatment of Tumours, in the region of Emilia Romagna (thus on patients who are not resident in Piedmont), for whom information on the histological type was available. This external validation confirmed a high sensitivity (95.6%), specificity (94.1%), and predictive values (99.9% and 80% as PPV and NPV, respectively). However, a major limitation relies on the size of the cohort of patients available, which was of 107 patients.

## 5. Conclusions

To our knowledge, this is the first Italian study on a validated procedure aimed at identifying cases of NSCLC and SCLC diagnosis from healthcare routinely collected data. 

Our validation based on a gold standard of 107 patients with diagnosis of lung cancer and histological confirmation showed 95% of accuracy, high sensitivity (95.6%), and specificity (94.1%).

Even if the validation was performed on a gold standard of only 107 patients, the operative characteristics of our procedure are comparable with those of the algorithm initially developed by Duh et al. [12] on a claims database, which achieved an accuracy of 92.1% with a sensitivity of 94.8% and a specificity of 81.1% [13].

The developed procedure is a promising easy-to-use tool to design subpopulation studies on lung cancer for risk stratification or for better planning resource allocation. The latter issue is crucial, since the characterization of the histologic type plays an increasingly pivotal role in the multidisciplinary approach in the diagnosis and management of lung carcinoma [4].

## Figures and Tables

**Table 1:** ATC codes of the drugs in the first line of treatment of NSCLC and SCLC according to the Italian Association of Medical Oncology Guidelines [17].

NSCLC		SCLC	
Drug	ATC Code	Drug	ATC Code
Crizotinib	L01XE16	Etoposide	L01CB01
Aletinib	L01XE36	Topotecan	L01XX17
Ceritinib	L01XE44	Carboplatin	L01XA02
Osimertinib	L01XE35	Cisplatin	L01XA01
Gefitinib	L01XE02		
Erlotinib	L01XE03		
Afatinib	L01XE13		
Nivolumab	L01XC17		
Pembrolizumab	L01XC18		
Bevacizumab	L01XC07		
Irinotecan	L01XX19		
Atezolizumab	L01XC32		
Durvalumab	L01XC28		
Platinum-based chemotherapy	L01XA		

ATC = Anatomical Therapeutic Chemical; NSCLC = non-small-cell lung cancer; SCLC = small-cell carcinoma.

**Table 2:** Procedures for radiotherapy and chemotherapy treatment considered for NSCLC/SCLC lung cancer characterization selected from the Outpatients Registry.

Procedure Description	Radiotherapy Treatment	Chemotherapy Treatment
Head CT with and without contrast		
Thoracic CT		
Thoracic CT with and without contrast		
Upper abdomen CT		
Upper abdomen CT with and without contrast		
Complete abdominal CT		
Complete abdominal CT with and without contrast		
MRI Brain and encephalic trunk		
MRI brain and encephalic trunk with and without contrast		
PET (quantitative)		
Total body PET		
Telecobalt therapy multiple fields, moving	X	
Teletherapy with linear accelerator with multiple fields or movement for 3D technique	X	
Teletherapy with linear accelerator with multiple fields or movement with modulation of intensity	X	
Teletherapy with linear accelerator fixed field	X	
Teletherapy with linear accelerator with multiple fields, moving	X	
Teletherapy with linear accelerator flash technique	X	
Radiotherapy with linear accelerator with MLC for IMRT static or dynamic multiple fields or moving	X	
Electron beam teletherapy with one or more fixed fields	X	
Total skin electron irradiation (TSEI/TSEBI)	X	
Injection or infusion of chemotherapy for tumor		X
Antitumoral therapy with infusion of drug		X
Antitumoral therapy with oral drugs or IM or subcutaneous injection		X

CT = Computer Tomography; PET = Positron Emission Tomography; IMRT = Intensity Modulated Radiotherapy; MLC = Multileaf Collimator; TSEI = Total Skin Electron Irradiation; TSEBI = Total Skin Electron Beam Irradiation; IM = Intramuscular.

**Table 3:** Procedure on respiratory system and radiotherapy treatment for NSCLC/SCLC lung cancer characterization to select from the Hospital Discharge Registry.

Procedure Code	Procedure Description	Procedure on Respiratory System	Radiotherapy Treatment
32 32.0–32.9	Excision of lung and bronchus	X	
33 33.0–33.9	Other operations on lung and bronchus	X	
34 34.0–34.9	Operations on the chest wall, pleura, mediastinum, and diaphragm	X	
92.21	Superficial radiation		X
92.22	Orthovoltage radiation		X
92.23	Radioisotopic teleradiotherapy		X
92.24	Teleradiotherapy using photons		X
92.25	Teleradiotherapy using electrons		X
92.26	Teleradiotherapy of other particulate radiation		X
92.27	Implantation or insertion of radioactive elements		X
92.28	Injection or instillation of radioisotopes		X
92.29	Other radiotherapeutic procedure		X
92.30	Stereotactic radiosurgery, not otherwise specified		X
92.31	Single source photon radiosurgery		X
92.32	Multi-source photon radiosurgery		X
92.33	Particulate radiosurgery		X
92.39	Stereotactic radiosurgery, not elsewhere classified		X

**Table 4:** Description of the cohort of patients with diagnosis of lung cancer in 2013–2017.

Variable	Alive at 31/12/2017	Dead at 31/12/2017	Total
	N = 8721	N = 22,706	N = 31,427
Sex, *N* (%)			
Females	2894 (33.2%)	6202 (27.3%)	9096 (.28.9%)
Males	5827 (66.8%)	16,504 (72.7%)	22,331 (71.1%)
Age, average (SD)	68.4 (10.4)	70.3 (10.5)	69.7 (10.5)

**Table 5:** Proportion of NSCLC and SCLC lung cancer cases estimated by the latent class model.

Latent Class Model	NSCLC, % (SE)	SCLC, % (SE)	AIC
Incident cases			
Model without information on radiotherapy treatment	84.8% (1.1%)	15.2% (1.1%)	12,292.81
Model with information on radiotherapy treatment	82.9% (1.4%)	17.1% (1.4%)	14,381.76
Prevalent cases			
Model without information on radiotherapy treatment	89.1% (1%)	10.9% (1%)	18,211.88
Model with information on radiotherapy treatment	80.2% (1.2%)	19.7% (1.2%)	21,131.83

SE = standard error.

## Data Availability

Code for data cleaning and analysis is available as GitHub repository at https://github.com/berkeley3/SCLC (accessed on: 31 May 2021).

## Supplementary Materials

The following are available online at https://www.mdpi.com/article/10.3390/ijerph18179076/s1, Table S1: Conditional probabilities of ATC code, outpatient procedures, and intervention respiratory system stratified by class on incident cases (without radiotherapy treatment). SCLC type was assigned to class 1; NSCLC type was assigned to class 2.
