# Supplementary material for: Incidence and Prevalence Analysis of Non-Small-Cell and Small-Cell Lung Cancer Using Administrative Data
Source: Int J Environ Res Public Health. 2021 Aug 28;18(17):9076. doi: 10.3390/ijerph18179076 (PMC8431612; doi:10.3390/ijerph18179076)
Supplement: Supplementary file 1 [file ijerph-18-09076-s001.zip › ijerph-1308555-supplementary.pdf]

Table S1. Conditional distribution of of ATC code, outpatient procedures and intervention respiratory system stratified by class (SCLC/NSCLC diagnosis) on incident cases from the latent class model without radiotherapy treatment. SCLC type was assigned to class 1; NSCLC type was assigned to class 2.

First ATC code	Class 1	Class 2	Second ATC	Class 1	Class 2	Outpatient procedure	Class 1	Class 2	Intervention on Respiratory system	Class 1	Class 2
L01CB01	0.33	0.00	L01XA01	0.12	0.00	87.03.1	0.84	0.56	No	0.05	0.08
L01XA01	0.23	0.06	L01XA02	0.22	0.00	87.41	0.02	0.14	Yes	0.95	0.92
L01XA02	0.38	0.10	L01XA03	0.00	0.00	87.41.1	0.05	0.16			
L01XA03	0.03	0.00	L01XC07	0.02	0.00	88.01.1	0.00	0.00			
L01XC07	0.01	0.00	L01XC17	0.36	0.00	88.01.2	0.00	0.00			
L01XC17	0.00	0.07	L01XC18	0.09	0.00	88.01.5	0.00	0.00			
L01XC18	0.01	0.03	L01XE02	0.04	0.00	88.01.6	0.00	0.01			
L01XE02	0.00	0.04	L01XE03	0.01	0.00	88.91.1	0.00	0.00			
L01XE03	0.00	0.01	L01XE13	0.00	0.01	88.91.2	0.02	0.02			
L01XE13	0.00	0.01	L01XE16	0.01	0.00	92.18.6	0.03	0.05			
L01XE16	0.01	0.01	L01XE35	0.00	0.01	92.24.2	0.01	0.00			
L01XE35	0.00	0.00	L01XE36	0.01	0.00	92.24.3	0.01	0.02			
L01XE36	0.00	0.00	L01XX17	0.01	0.00	92.24.B	0.00	0.00			
L01XX17	0.00	0.00	L01XX19	0.00	0.00	99.25	0.00	0.00			
L01XX19	0.00	0.00	none	0.11	0.98	99.25.4	0.03	0.02			
none	0.00	0.67				99.25.5	0.00	0.01			
